# Theaflavins counteract free fatty acid-driven oxidative-inflammatory injury in endothelial cells through Nrf2-NF-κB axis

**DOI:** 10.3389/fimmu.2026.1807168

**Published:** 2026-06-09

**Authors:** Yufei Zhao, Biao Qu

**Affiliations:** 1 Cardiovascular Department for Gerontism, The Second Affiliated Hospital of Anhui Medical University, Hefei, China; 2 Department of Clinical Pharmacology, The Second Hospital of Anhui Medical University, Hefei, China

**Keywords:** endothelial injury, inflammatory response, lipotoxicity, Nrf2-NF-κB axis, oxidative stress, theaflavins, vascular endothelial cells

## Abstract

**Background:**

Chronically elevated circulating free fatty acids (FFAs) are key drivers of lipotoxic injury by triggering a self-amplifying oxidative stress-inflammation cascade, thereby contributing to metabolic and cardiovascular diseases. Vascular endothelial dysfunction represents an early and causal event in this process. Although black tea-derived theaflavins (TFs) possess antioxidant and anti-inflammatory properties, the subtype-specific and tissue-parallel effects of TFs against FFA-induced oxidative-inflammatory injury remain incompletely understood.

**Methods:**

FFA-induced lipotoxicity models were established in vascular endothelial cells and hepatocytes to systematically compare the protective effects of four major TF subtypes: theaflavin (TF1), theaflavin-3-gallate (TF2A), theaflavin-3′-gallate (TF2B), and theaflavin-3, 3′-digallate (TFDG). Antioxidant capacity was assessed using both cell-free systems and oxidant-stimulated cellular models. Mechanistic studies were conducted to determine the involvement of the Nrf2-NF-κB axis.

**Results:**

All TF subtypes significantly attenuated FFA-induced ROS overproduction, lipid peroxidation, and inflammatory mediator release in both endothelial cells and hepatocytes, whereas no major changes in intracellular lipid accumulation were observed. Among the four subtypes, TF1 consistently exhibited the strongest and most reproducible cytoprotective effects. Further analyses demonstrated that TFs, particularly TF1, restored cellular redox homeostasis not only through direct free radical scavenging but, more importantly, through activation of endogenous antioxidant defenses. Transcriptomic and bioinformatics analyses indicated that TF1 enhanced antioxidant-related gene expression while suppressing inflammatory genes, with enrichment of oxidative stress-, atherosclerosis-, and NF-κB-related pathways and Nrf2 appearing as a key node. Molecular docking predicted a favorable TF1-Keap1 interaction. Consistently, TF1 promoted Nrf2 nuclear translocation and HO-1 expression, restored SOD2 and GPX1 expression, reduced mitochondrial ROS, and attenuated IκBα phosphorylation and NF-κB p65 nuclear accumulation. Silencing Nrf2 markedly weakened these protective effects, supporting an important role of Nrf2 in TF1-mediated endothelial protection.

**Conclusion:**

TFs act as natural modulators of FFA-driven oxidative-inflammatory injury, with TF1 emerging as the most potent subtype. TF1 protects against FFA-induced endothelial dysfunction partly through an Nrf2-associated mechanism involving enhanced antioxidant responses and attenuation of NF-κB-related inflammatory signaling.

## 1 Introduction

The prevalence of metabolic diseases and associated cardiovascular disorders constitutes a major global public health challenge (1). A shared biochemical hallmark is the chronic elevation of circulating free fatty acids (FFAs), which functions not merely as a marker of metabolic imbalance but also as a direct mediator of lipotoxic tissue injury (2). The vascular endothelium and the liver are two major targets of systemic FFA overload, representing distinct but interconnected facets of lipotoxicity. As the first vascular interface exposed to circulating FFAs, the endothelium is especially susceptible to lipotoxic stress, which disrupts barrier integrity, impairs vasoregulatory function, and promotes a pro-atherogenic phenotype that contributes to atherosclerosis initiation and progression (3–5). In contrast, the liver is the principal site of FFA uptake and metabolic processing, and excessive FFA influx drives lipid accumulation and hepatocellular stress, thereby promoting the development and progression of metabolic dysfunction-associated fatty liver disease (MAFLD) (6). Thus, endothelial cells and hepatocytes capture complementary aspects of systemic lipotoxicity and together provide complementary and biologically relevant models for evaluating the impact of FFA overload across key vascular and metabolic tissues.

At the cellular level, FFA-induced lipotoxicity is largely mediated by a coupled oxidative stress-inflammation cascade. When FFA influx exceeds the capacity for mitochondrial β-oxidation, lipid storage, and detoxification, surplus fatty acids and toxic lipid intermediates accumulate, impairing mitochondrial function and redox balance. This enhances electron leakage from the respiratory chain and results in reactive oxygen species (ROS) overproduction and oxidative damage (7). ROS not only damage macromolecules directly but also initiate lipid peroxidation, generating reactive lipid products that further amplify oxidative injury (8). ROS and lipid-derived stress signals also activate redox-sensitive inflammatory pathways, particularly NF-κB signaling, thereby increasing pro-inflammatory mediator expression (9). This self-reinforcing FFA-ROS-inflammation axis links lipid overload to cellular dysfunction. In endothelial cells, it reduces nitric oxide bioavailability, promotes endothelial activation, and accelerates vascular injury (10, 11). In hepatocytes, it aggravates lipid metabolic disorder, inflammatory signaling, and hepatocellular damage. Together, these mechanisms highlight oxidative-inflammatory signaling as a key target for mitigating FFA-induced lipotoxicity.

Dietary polyphenols are increasingly studied as safe modulators of oxidative stress and inflammation. Black tea is rich in theaflavins (TFs), benzotropolone-containing polyphenols formed during fermentation (12, 13). Among >20 TFs, theaflavin (TF1), theaflavin−3−gallate (TF2A), theaflavin−3′−gallate (TF2B), and theaflavin−3, 3′−digallate (TFDG) are the major subtypes and differ in galloyl substitution, which can shape bioactivity and bioavailability. Epidemiological and experimental studies suggest that black tea intake is associated with metabolic resilience and vascular benefits, effects that are likely attributable to TFs (14–17). In vascular systems, TFs have been reported to improve endothelial function, inhibit low−density lipoprotein oxidation, and attenuate atherosclerotic lesion formation (18). In hepatic contexts, TFs have been shown to reduce lipid peroxidation and suppress inflammatory responses across diverse experimental models (19). Despite these encouraging findings, existing evidence remains limited by a predominant focus on total theaflavin mixtures or single TF subtypes, thereby obscuring potential structure-activity relationships. In addition, endothelial and hepatic effects are rarely compared under matched lipotoxic conditions, obscuring whether TFs exert coordinated protection across “gatekeeper” tissues. Moreover, mechanistic studies are often fragmentary, emphasizing isolated antioxidant or anti−inflammatory endpoints without systematically delineating the integrated upstream-downstream signaling events.

Addressing these limitations is critical for advancing both mechanistic insight and translational relevance. Building on the central role of FFA−driven oxidative-inflammatory injury in vascular and hepatic dysfunction, the present study performs a systematic, side−by−side comparison of the four primary TF subtypes in the context of FFA−induced lipotoxicity in vascular endothelial cells and hepatocytes. Specifically, we examine their relative protective effects under lipotoxic conditions, assess whether these effects are primarily associated with attenuation of the oxidative stress-inflammation cascade, and further investigate the mechanism underlying the activity of the lead subtype. By integrating subtype-level comparison, cross-tissue validation, and mechanistic interrogation, this study aims to clarify the biological basis of theaflavin-mediated protection against FFA-induced lipotoxic injury and to provide a stronger rationale for their potential use as diet-derived modulators of lipotoxic stress.

## 2 Materials and methods

### 2.1 Chemicals

TF1 (purity: 99.28%, lot No. ST9H9BC1A601), TF2A (purity: 99.33%, lot No. ST9H9BC1A602), TF2B (purity: 99.89%, lot No. ST9H9BC1A5FF), and TFDG (purity: 99.20%, lot No. ST9H9BC1EBB6), hydrogen peroxide (H_2_O_2_; Cat No. H1009), sodium palmitate (Cat No. P9767), ascorbic acid (VC; Cat No. PHR1008), 3-(4, 5-dimethylthiazol-2-yl)-2, 5-diphenyltetrazolium bromide (MTT; Cat No. 475989), fatty acid-free bovine serum albumin (BSA; Cat No. B2064), and H_2_DCF-DA (Cat No. D6883) were purchased from Sigma-Aldrich (St. Louis, MO, USA). Dulbecco’s modified Eagle medium (DMEM), penicillin-streptomycin antibiotic solution, fetal bovine serum (FBS), and trypsin-EDTA were obtained from Gibco BRL (Grand Island, NY, USA). siRNAs of si-NC and si-Nrf2 were purchased from Ribobio (Guangzhou, China). Recombinant human KEAP1 protein (Cat No. Ag0779) was purchased from Proteintech (Wuhan, China). Primary antibodies against Nrf2 (Cat No. 12721), HO-1(Cat no: 43966), SOD2 (Cat no: 13141), GPX1(Cat no: 3286), IκBα (Cat no: 4812), NF-κB p65 (Cat no: 8242), GAPDH (Cat no: 2118) and Histone H3 (Cat no: 4499)were supplied by Cell Signaling Technology (Beverly, MA, USA). Horseradish peroxidase (HRP)-conjugated secondary antibody (Cat no: 7074) was provided by Santa Cruz Biotechnology (Santa Cruz, CA, USA).

### 2.2 Cell culture and experimental design

EA.hy926 and HepG2 cells (American Type Culture Collection; Bethesda, MD, USA) were used as *in vitro* models of vascular endothelial cells and hepatocytes, respectively. These cell lines are well-established and widely used for studying endothelial dysfunction, hepatocellular injury, and FFA-induced lipotoxic stress under controlled experimental conditions. However, EA.hy926 is a hybrid endothelial cell line, and HepG2 cells exhibit altered lipid metabolism compared with primary hepatocytes; thus, the findings should be interpreted within the limitations of these models. Both cell lines were cultured in DMEM supplemented with 10% FBS, 100 U/mL penicillin, and 100 μg/mL streptomycin in a humidified atmosphere containing 5% CO_2_ at 37 °C. For lipotoxicity experiments, sodium palmitate was conjugated with fatty acid-free BSA in PBS to prepare a 5 mM stock solution and then diluted in culture medium to a final concentration of 0.5 mM. Cells were exposed to 0.5 mM palmitate for 24 h to induce FFA-mediated lipotoxic injury, while control cells received the corresponding BSA vehicle. For direct oxidative injury experiments, H_2_O_2_ was freshly prepared in DMEM and used at a final concentration of 0.5 mM. In all experiments, cells were pretreated with TFs (10 μM) in DMEM for 2 h. Subsequently, cells were exposed to palmitate or H_2_O_2_ in the presence or absence of TFs for an additional 24 h.

### 2.3 Cell viability assay

Cell viability was evaluated using the MTT assay. Cells were seeded into 96-well plates at a density of 1.0 × 10_4_ cells per well and allowed to attach overnight. After the indicated treatments, 10 μL of MTT solution (5 mg/mL) was added to each well and incubated for 4 h at 37 °C. The culture medium was carefully removed, and the resulting formazan crystals were dissolved in 150 μL DMSO. Absorbance was measured at 570 nm using a microplate reader (BioTek, Winooski, VT, USA). Cell viability was expressed as a percentage relative to the control group.

### 2.4 Measurement of cellular injury markers

After the indicated treatments, for EA.hy926 cells, endothelial injury was assessed by measuring nitric oxide (NO) production and lactate dehydrogenase (LDH) release using commercial kits (Biovision, CA, USA). In HepG2 cells, liver injury was evaluated by measuring ALT and AST activities using assay kits (Nanjing Jiancheng Bioengineering Institute, Nanjing, China). All assays were conducted according to the manufacturers’ instructions.

### 2.5 Lipid accumulation, oxidative stress and inflammatory response analysis

Intracellular lipid accumulation was visualized by Oil Red O staining. Intracellular FFA levels were measured using a nonesterified fatty acid assay kit (Nanjing Jiancheng Bioengineering Institute). Intracellular ROS and mitochondrial ROS were detected using H_2_DCF-DA and MitoSOX Red, respectively. Fluorescence images were acquired under identical settings, and mean fluorescence intensity was quantified using Image-Pro Plus 6.0 software with consistent threshold and measurement parameters across all images. Lipid peroxidation and antioxidant status were evaluated by measuring malondialdehyde (MDA) levels and the activities of superoxide dismutase (SOD) and glutathione peroxidase (GPx) using commercial assay kits (Nanjing Jiancheng Bioengineering Institute). Inflammatory mediators in culture supernatants, including the pro-inflammatory cytokines TNF-α and IL-1β, as well as VCAM-1, were quantified using ELISA kits (Proteintech, Wuhan, China) according to the manufacturers’ instructions. VCAM-1 is an endothelial adhesion molecule and was included as an indicator of endothelial inflammatory activation and leukocyte adhesion-related dysfunction.

### 2.6 Determination of chemical antioxidant capacity of TFs

The antioxidant capacity of TFs was evaluated via ABTS radical-scavenging, DPPH radical-scavenging, and hydroxyl radical (OH)-scavenging assays using commercial kits (Nanjing Jiancheng Bioengineering Institute, Nanjing, China) following the manufacturer’s instructions. The results were expressed as half-maximal effective concentration (EC_50_, μg/mL) from three replicated measurements. Vitamin C (VC) was used as a positive control.

### 2.7 RNA sequencing, bioinformatics analysis, and molecule docking

Total RNA was extracted using TRIzol reagent (Invitrogen, USA). RNA quantity, purity, and integrity were assessed, and qualified samples were used for library preparation. Poly(A)+ RNA was enriched, fragmented, and reverse-transcribed into cDNA. RNA-seq was performed using three biological replicates per group. Strand-specific libraries were prepared through second-strand synthesis with dUTP incorporation, followed by adapter ligation, size selection, UDG treatment, and PCR amplification. Libraries with an average insert size of 300 ± 50 bp were sequenced on an Illumina NovaSeq 6000 platform (Illumina, USA) by LC-Bio Technology Co., Ltd. (Hangzhou, China). Raw reads were processed with fastp for adapter trimming and quality filtering. Clean reads were aligned to GRCh38, followed by transcript assembly and merging. Gene expression levels were quantified as FPKM. Differentially expressed genes (DEGs) were defined as genes with a fold change > 2 or < 0.5 and an adjusted P value (false discovery rate, FDR) < 0.05, with multiple testing correction performed using the Benjamini-Hochberg method. Gene Ontology (GO)/KEGG pathway enrichment and gene set enrichment analysis (GSEA) were carried out for the DEGs. Molecular docking was performed to evaluate the interaction between TF1 and the target protein. The 3D structure of TF1 was generated from its ChemDraw-derived chemical structure and energy-minimized before docking. The crystal structure of the target protein was obtained from the RCSB Protein Data Bank. Docking was conducted using the CB-Dock2 online server, which combines curvature-based cavity detection with AutoDock Vina-based blind docking (20). Putative binding pockets were automatically identified, and the docking pose with the lowest Vina score was selected as the optimal binding mode for subsequent analysis.

### 2.8 Quantitative real-time PCR and western blot

To validate gene expression, total RNA was extracted from treated cells using standard procedures. qRT-PCR was performed using Power SYBR Green Master Mix (Thermo Fisher, CA, USA) on a real-time PCR system. The fold-change in gene expression was calculated using the comparative 2^−ΔΔCt^ method. Primer sequences are listed in **Supplementary Table 1**. For western blot analysis, total proteins were prepared using lysis buffer containing protease and phosphatase inhibitors, while nuclear fractions were prepared using a nuclear extraction procedure according to the manufacturer’s instructions. Equal amounts of protein were separated on 12% SDS-PAGE and transferring them to PVDF membranes. Membranes were probed with specific primary antibodies against Nrf2, HO-1, SOD2, GPX1, IκBα, p-IκBα, NF-κB p65, GAPDH and Histone H3, followed by HRP-conjugated secondary antibodies. Bands were visualized using ECL reagent and quantified with ImageJ software. GAPDH was used as the internal control for total proteins, and Histone H3 was used as the internal control for nuclear proteins.

### 2.9 siRNA transfection

EA.hy926 cells were seeded at 1×10^5^ cells/well in 6-well plates and cultured in serum-free DMEM for 24 h prior to transfection. Transfection with si-Nrf2 or si-NC was performed using a transfection reagent according to the manufacturer’s instructions. The sequences of siRNAs were provided in **Supplementary Table 2**. Cells were incubated for 24 h post-transfection, and transfection efficiency was verified by Western blot before proceeding with further experiments.

### 2.10 Statistical analysis

All data were expressed as means ± standard deviation (SD) from three independent experiments (n = 3). Statistical analyses of the data were performed using GraphPad Prism version 8.0. Comparisons between groups were performed using one-way analysis of variance (ANOVA) followed by Tukey’s *post-hoc* test, with p < 0.05 considered statistically significant.

## 3 Results

### 3.1 Cytotoxicity of TFs in EA.hy926 and HepG2 cells

**Figure 1:**
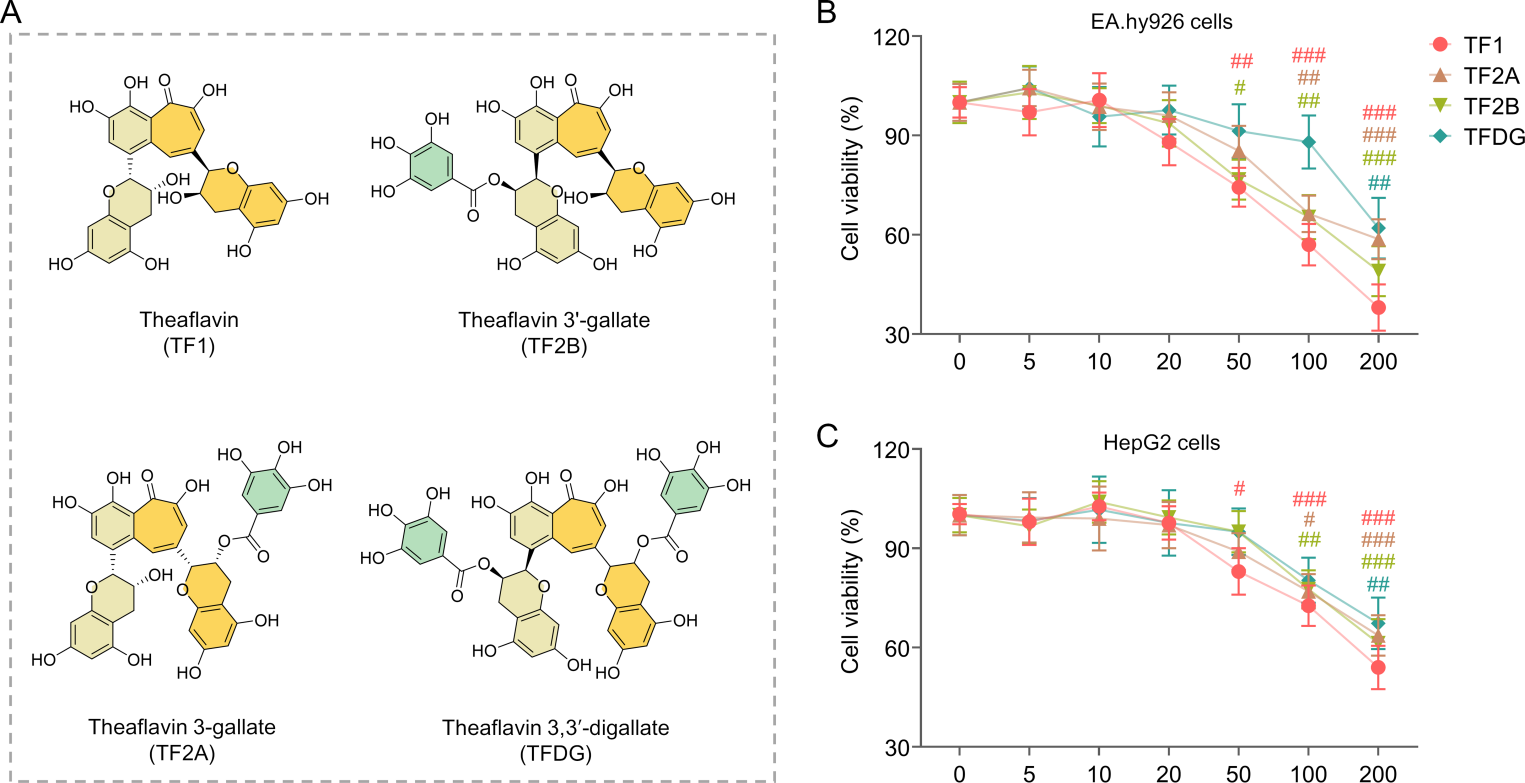
Chemical structures and cytotoxicity of TFs. **(A)** Chemical structures of the four TFs; **(B, C)** Effects of different concentrations of the four TFs on the viability of EA.hy926 cells **(B)** and HepG2 cells **(C)**. ^#^p < 0.05, ^##^p < 0.01, and ^###^p < 0.001 *vs.* Control group (0 µM). TFs, theaflavins.

The chemical structures of the four TFs were illustrated in **Figure 1A**, and these compounds share a benzotropolone core but differ in the number and position of their galloyl groups. To determine non-cytotoxic concentrations for subsequent experiments, the effects of four TFs on cell viability were evaluated in EA.hy926 and HepG2 cells. As shown in **Figures 1B, C**, TFs affected cell viability in a concentration-dependent manner. Treatment with concentrations ranging from 5 to 20 µM did not significantly reduce cell viability compared to the control group. In contrast, treatment with ≥ 50 µM resulted in a significant decrease in viability for certain monomers, while higher concentrations (100 and 200 µM) induced pronounced cytotoxicity in all groups (p < 0.001). Based on these findings, 10 µM was selected as a non-cytotoxic concentration for subsequent experiments.

### 3.2 Protective effects of TFs against FFA-induced injury in EA.hy926 and HepG2 cells

**Figure 2:**
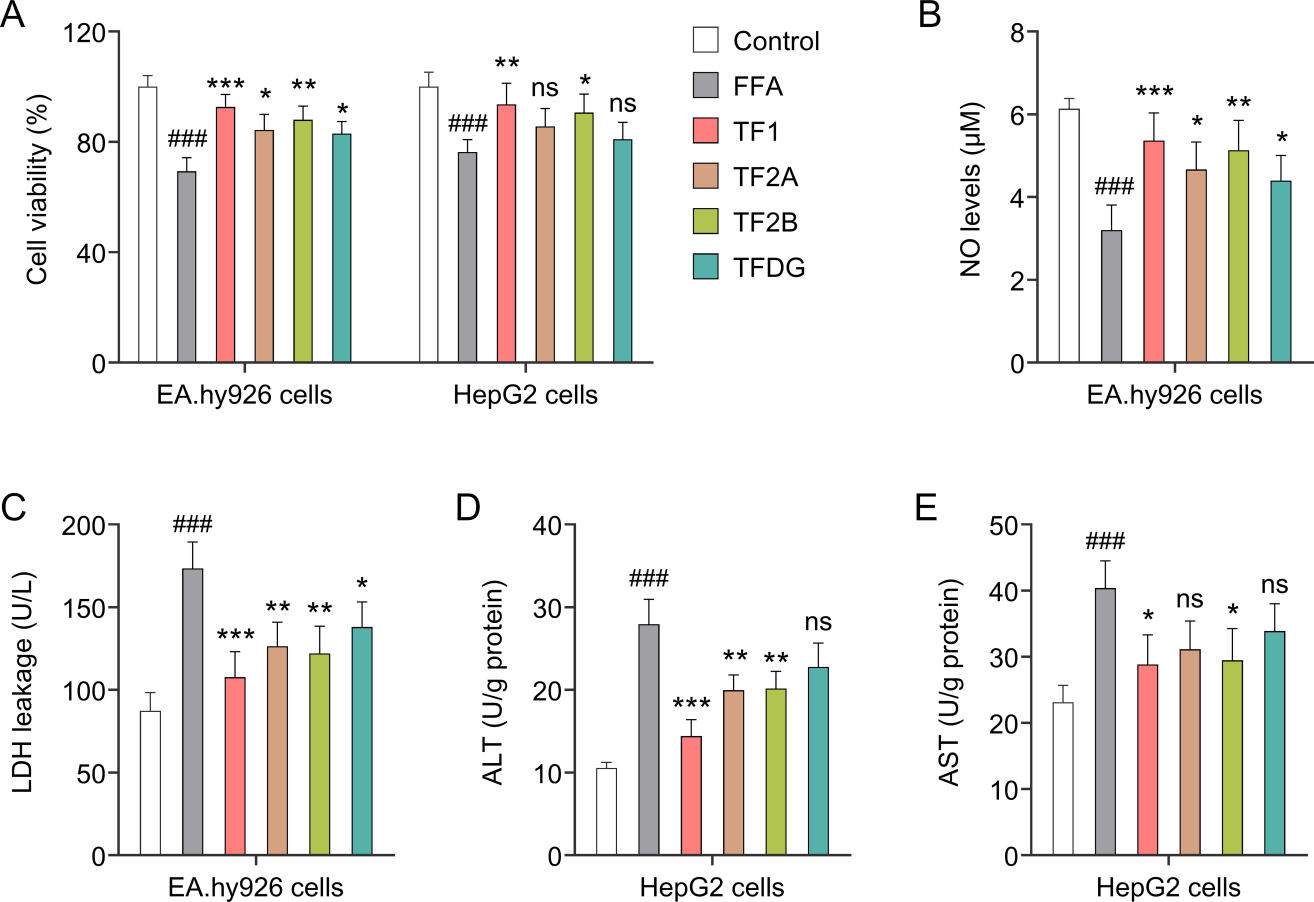
Protective effects of TFs against FFA-induced cellular injury. **(A)** Effects of TFs on cell viability of EA.hy926 and HepG2 cells treated with FFA; **(B, C)** NO production **(B)** and LDH leakage **(C)** in EA.hy926 cells; **(D, E)** Activities of ALT **(D)** and AST **(E)** in HepG2 cells. ^###^p < 0.001 *vs.* Control group; ^∗^p < 0.05, ^∗∗^p < 0.01, ^∗∗∗^p < 0.001, and ns (not significant) *vs.* FFA group. TFs, theaflavins; TF1, theaflavin; TF2A, theaflavin-3-gallate; TF2B, theaflavin-3′-gallate; TFDG, theaflavin-3, 3′-digallate; FFA, free fatty acid; NO, nitric oxide; LDH, lactate dehydrogenase.

FFA treatment significantly reduced the viability of both EA.hy926 and HepG2 cells (p < 0.001, **Figure 2A**), confirming the successful induction of lipotoxicity. However, pretreatment with TFs effectively mitigated this damage. TF1 exhibited the most potent protection, restoring EA.hy926 viability to 92.7% of the control and also significantly improving HepG2 survival. In contrast, TFDG did not show a statistically significant protective effect in HepG2 cells under the present experimental conditions. Regarding cell-specific injury markers, TFs reversed the FFA-induced decline in NO production (**Figure 2B**) and elevation of LDH leakage (**Figure 2C**) in EA.hy926 cells, with TF1 showing the highest efficacy (p < 0.001 for both NO level and LDH leakage). Similarly, in HepG2 cells, TF1 demonstrated the strongest hepatoprotective effect, reducing ALT (p < 0.001, **Figure 2D**) and AST (p < 0.001, **Figure 2E**) levels most significantly, followed by TF2A and TF2B, whereas TFDG showed a weaker effect that did not reach statistical significance. Collectively, these results suggested that TFs protected against FFA-induced cellular injury in both endothelial and hepatic cells, with TF1 exhibiting the most consistent and potent protective activity.

### 3.3 TFs attenuated the FFA-ROS-inflammation axis without major changes in lipid accumulation

**Figure 3:**
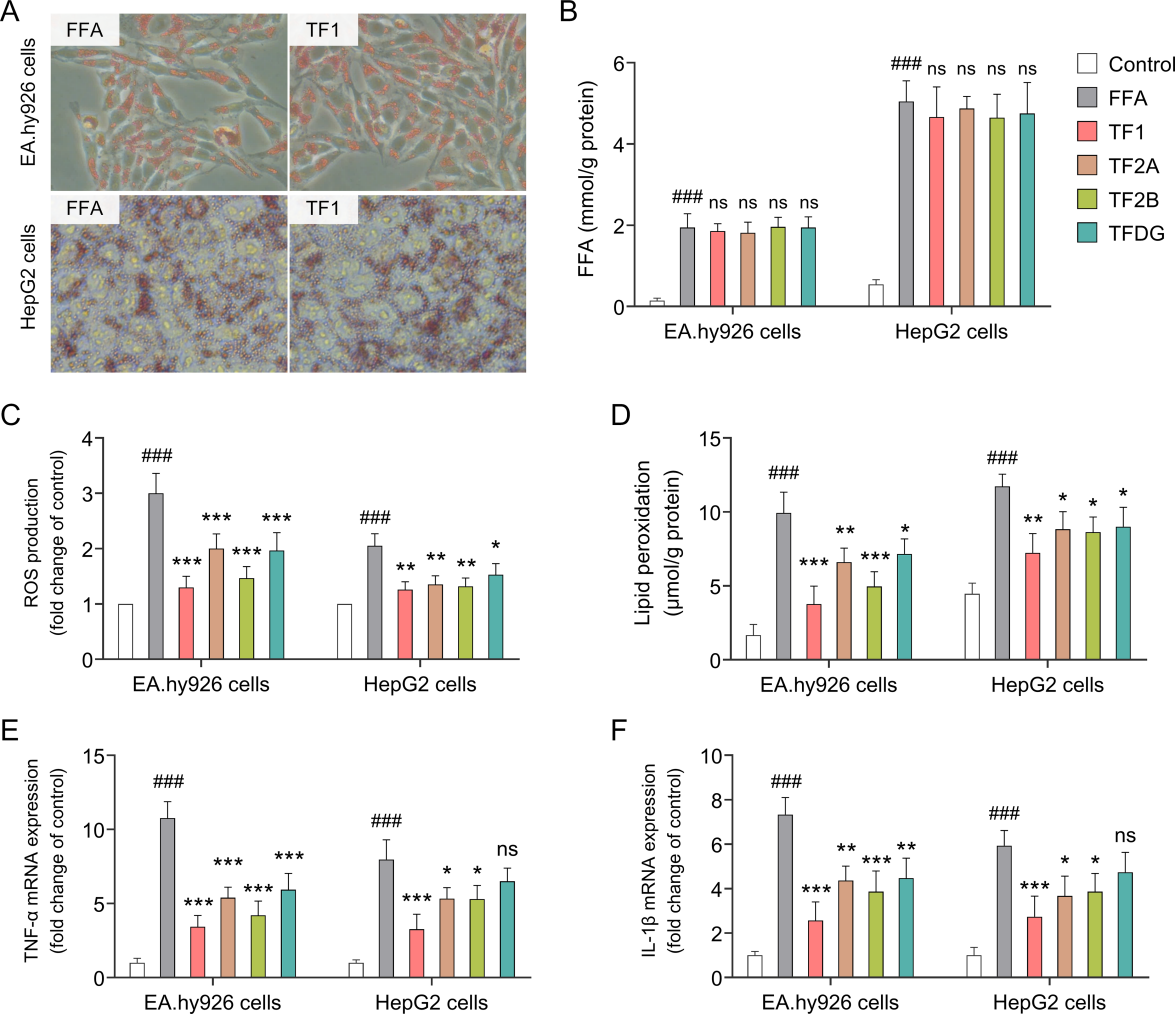
TFs attenuated oxidative stress and inflammation without reducing lipid accumulation. **(A)** Oil red O staining images of EA.hy926 and HepG2 cells; **(B)** quantification of intracellular FFA levels; **(C, D)** assessment of oxidative stress markers: lipid peroxidation levels **(C)** and relative ROS production **(D)**; **(E, F)** mRNA expression levels of pro-inflammatory cytokines TNF-α **(E)** and IL-1β **(F)**. ^###^p < 0.001 *vs.* Control group; ^∗^p < 0.05, ^∗∗^p < 0.01, ^∗∗∗^p < 0.001, and ns (not significant) *vs.* FFA group. TFs, theaflavins; TF1, theaflavin; TF2A, theaflavin-3-gallate; TF2B, theaflavin-3′-gallate; TFDG, theaflavin-3, 3′-digallate; FFA, free fatty acid; ROS, reactive oxygen species.

To investigate whether the cytoprotective effects of TFs were associated with altered lipid accumulation, we first examined intracellular lipid droplets after FFA exposure. As shown in **Figure 3A**, FFA treatment markedly increased lipid droplets in both EA.hy926 and HepG2 cells. Representative images from the TF1 group were shown, as the other TF treatments exhibited similar patterns. Quantitative analysis further confirmed that intracellular FFA levels were significantly increased in the FFA group (**Figure 3B**, p < 0.001). However, TF treatment did not produce major changes in intracellular lipid droplet accumulation or FFA levels compared with the FFA group. These findings suggest that, under the present experimental conditions, the cytoprotective effects of TFs were not accompanied by a marked reduction in overall intracellular lipid accumulation. Given that high lipid loads trigger oxidative stress, we examined the ROS-lipid peroxidation axis. FFA treatment caused a surge in lipid peroxidation (p < 0.001, **Figure 3C**) and ROS production (p < 0.001, **Figure 3D**). In contrast to the lipid accumulation results, TFs exerted significant inhibition on these oxidative events. Specifically, TF1 exhibited the most potent antioxidant activity, reducing lipid peroxidation and ROS levels by over 50% in both EA.hy926 and HepG2 cells (p < 0.001). Since oxidative stress is a potent trigger for inflammatory responses, we further evaluated the mRNA expression of key pro-inflammatory cytokines. FFA exposure significantly upregulated TNF-α (**Figure 3E**) and IL-1β (**Figure 3F**) mRNA levels. Consistent with the antioxidant results, TFs effectively suppressed this inflammatory cascade. TF1 and TF2A showed robust anti-inflammatory effects (p < 0.001), reversing cytokine expression to near-control levels. However, TFDG did not significantly reduce TNF-α or IL-1β expression in HepG2 cells under the present conditions. Together, these data indicated that TFs treatment were associated with attenuation of oxidative stress and inflammatory responses in FFA-treated cells, despite no major changes in lipid accumulation. Among them, TF1 served as the most potent agent for blocking FFA-induced ROS-inflammation cascade.

### 3.4 Direct antioxidant capacity of TFs in cell-free systems

**Figure 4:**
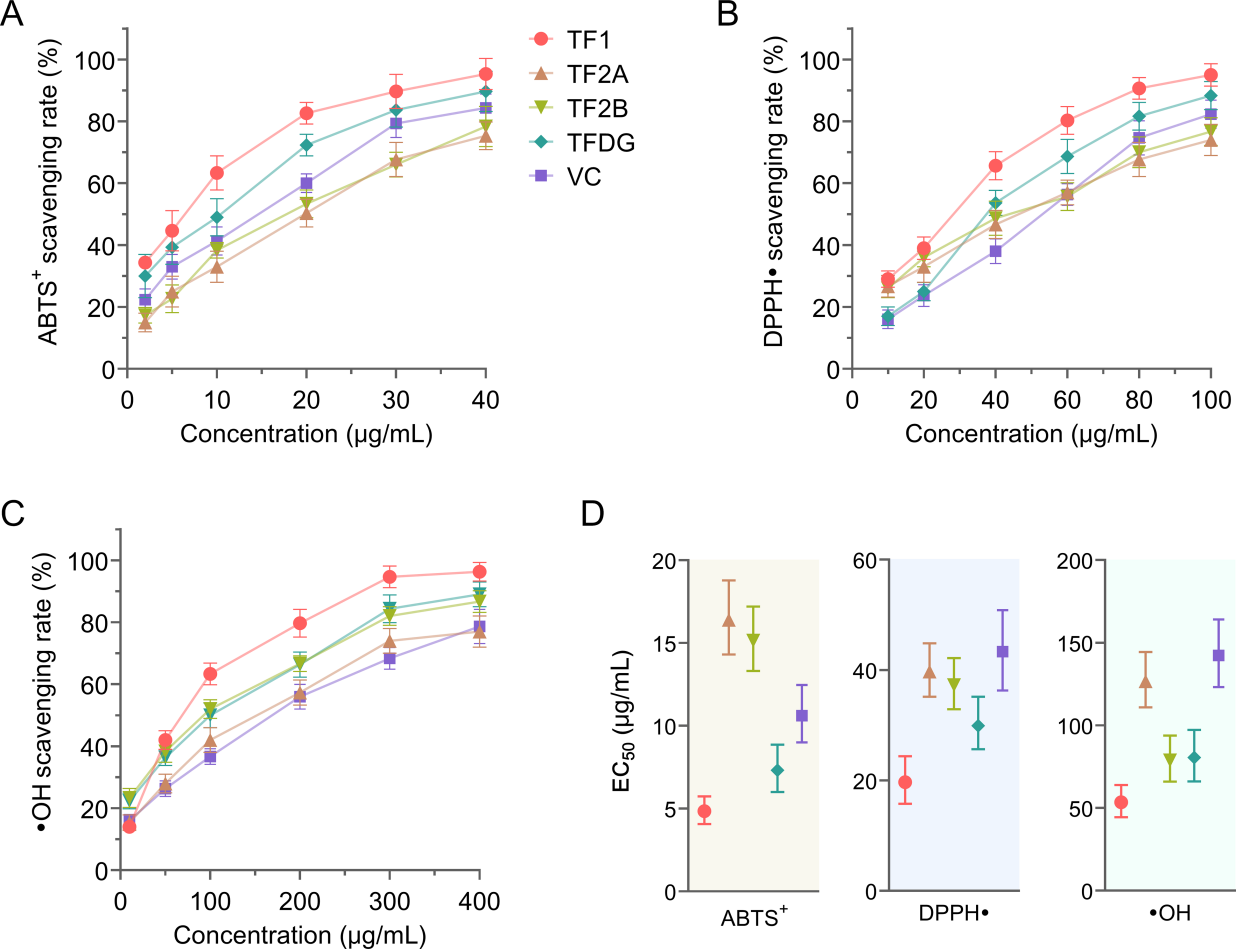
Direct antioxidant activities of TFs in cell-free systems. **(A–C)** scavenging rates of TFs and VC against ABTS+ **(A)**, DPPH· **(B)**, and ·OH **(C)** radicals at various concentrations; **(D)** comparison of the EC_50_ values for the three antioxidant assays. Lower EC_50_ values indicate higher antioxidant potency. TFs, theaflavins; TF1, theaflavin; TF2A, theaflavin-3-gallate; TF2B, theaflavin-3′-gallate; TFDG, theaflavin-3, 3′-digallate; VC, ascorbic acid; EC_50_, half-maximal effective concentration.

To determine whether the observed cytoprotective effects were attributable to the intrinsic radical-scavenging capability of TFs, their direct antioxidant activities were evaluated using ABTS, DPPH, and OH assays in cell-free systems. As illustrated in **Figures 4A–C**, all four TF monomers exhibited concentration-dependent scavenging activities. TF1 consistently demonstrated the highest potency, yielding the lowest EC_50_ values across all assays (e.g., 4.85 ± 0.89 μg/mL for ABTS, 19.70 ± 3.92 μg/mL for DPPH, 53.46 ± 10.39 μg/mL for OH; **Figure 4D**), which aligns with its superior cellular efficacy. Notably, a divergence was observed regarding TFDG: while it displayed stronger chemical scavenging capacity than TF2A and TF2B (lower EC_50_), it was less effective in protecting cells against FFA-induced injury. This discrepancy implied that the cytoprotective mechanism of TFs extended beyond direct chemical neutralization, likely involving the modulation of intracellular antioxidant defense systems.

### 3.5 TFs protected against H_2_O_2_-induced oxidative injury by restoring cellular antioxidant system

**Figure 5:**
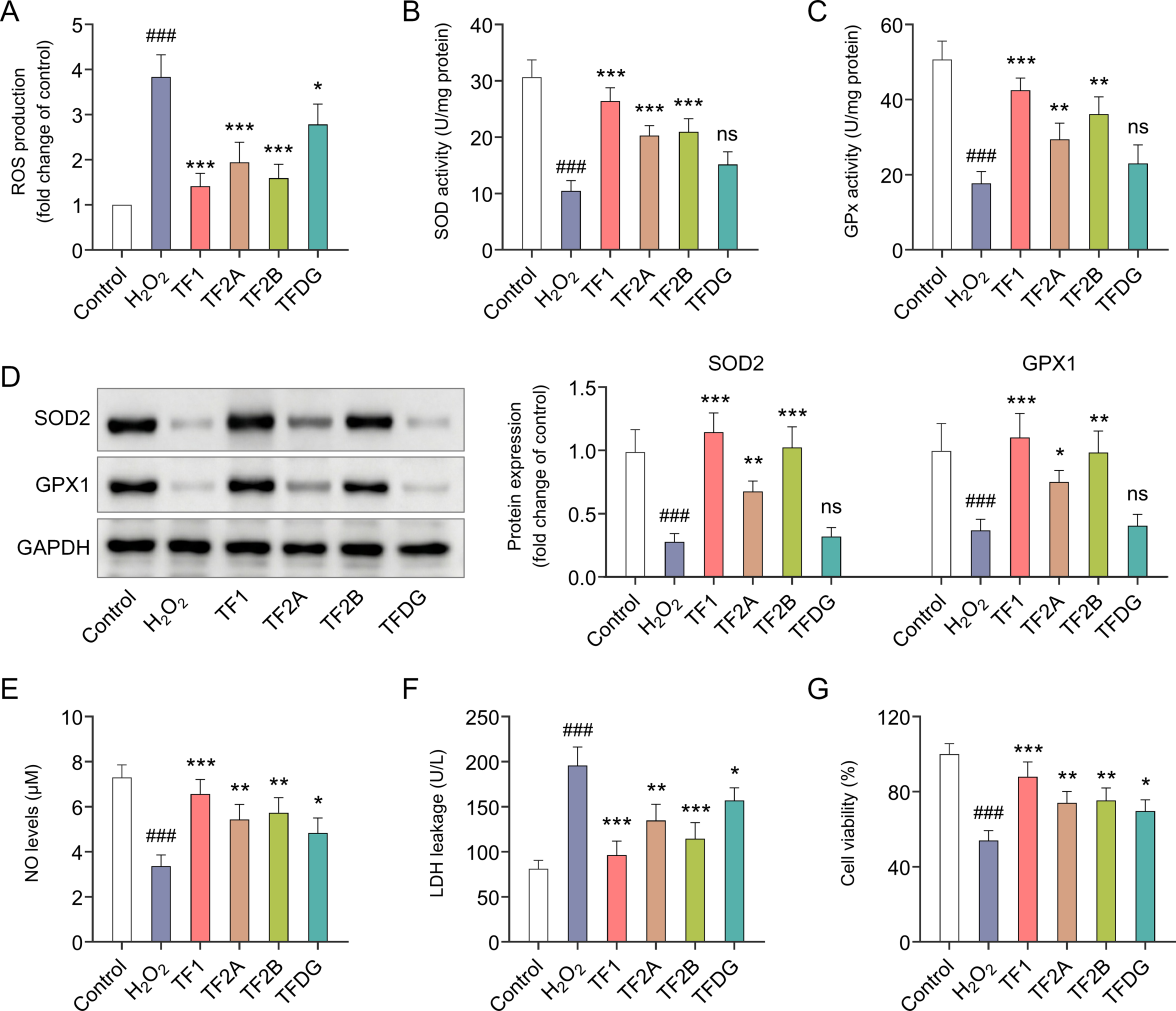
TF1 exerted superior protective effects on EA.hy926 cells by enhancing cellular antioxidant system in an H_2_O_2_-induced model. **(A)** Intracellular ROS production levels; **(B, C)** the enzymatic activities of SOD **(B)** and GPx **(C)**; **(D)** protein expression of SOD2 and GPX1; **(E, F)** measurement of NO levels **(E)** and LDH leakage **(F)**; **(G)** measurement of cell viability. ^###^p < 0.001 *vs.* Control group; ^∗^p < 0.05, ^∗∗^p < 0.01, ^∗∗∗^p < 0.001, and ns (not significant) *vs.* H_2_O_2_​ group. TF1, theaflavin; TF2A, theaflavin-3-gallate; TF2B, theaflavin-3′-gallate; TFDG, theaflavin-3, 3′-digallate; ROS, reactive oxygen species; SOD, superoxide dismutase; GPx, glutathione peroxidase; SOD2, superoxide dismutase 2; GPX1, glutathione peroxidase 1; NO, nitric oxide; LDH, lactate dehydrogenase.

To further establish the link between antioxidant capacity and cellular protection, an H_2_O_2_-induced oxidative stress model was established in EA.hy926 cells. This model was used as a complementary oxidative stress system to evaluate the antioxidant effects of TFs under a simplified ROS-driven condition. H_2_O_2_ exposure caused a pronounced redox imbalance, marked by a significant increase in intracellular ROS levels (p < 0.001; **Figure 5A**) and a concomitant reduction in SOD and GPx activities (p < 0.001; **Figures 5B, C**). Strikingly, the antioxidative efficacy of theaflavins in this cellular model paralleled that observed in the FFA-induced model (TF1 > TF2A/B > TFDG), diverging from the intrinsic chemical antioxidant potential. TF1 most effectively reduced ROS accumulation and restored SOD and GPx activities (**Figures 5A–C**), followed by TF2A and TF2B, whereas TFDG exhibited only limited effects in this model. Western blot analysis revealed a clear structure-activity relationship at the protein level. TF1 markedly upregulated the expression of SOD2 and GPX1, while TFDG did not produce comparable changes relative to the model group (**Figure 5D**). Consistent with enhanced antioxidant defense, TF1 significantly alleviated endothelial dysfunction, as evidenced by restored NO production (**Figure 5E**), reduced LDH release (**Figure 5F**), and improved cell viability (**Figure 5G**). Together, these findings suggest that the superior cellular protection afforded by TF1 was closely linked to its ability to reinforce endogenous antioxidant defenses, rather than to direct radical scavenging alone.

### 3.6 TF1 mitigated FFA-induced endothelial cell injury with concomitant suppression of oxidative stress and inflammation

**Figure 6:**
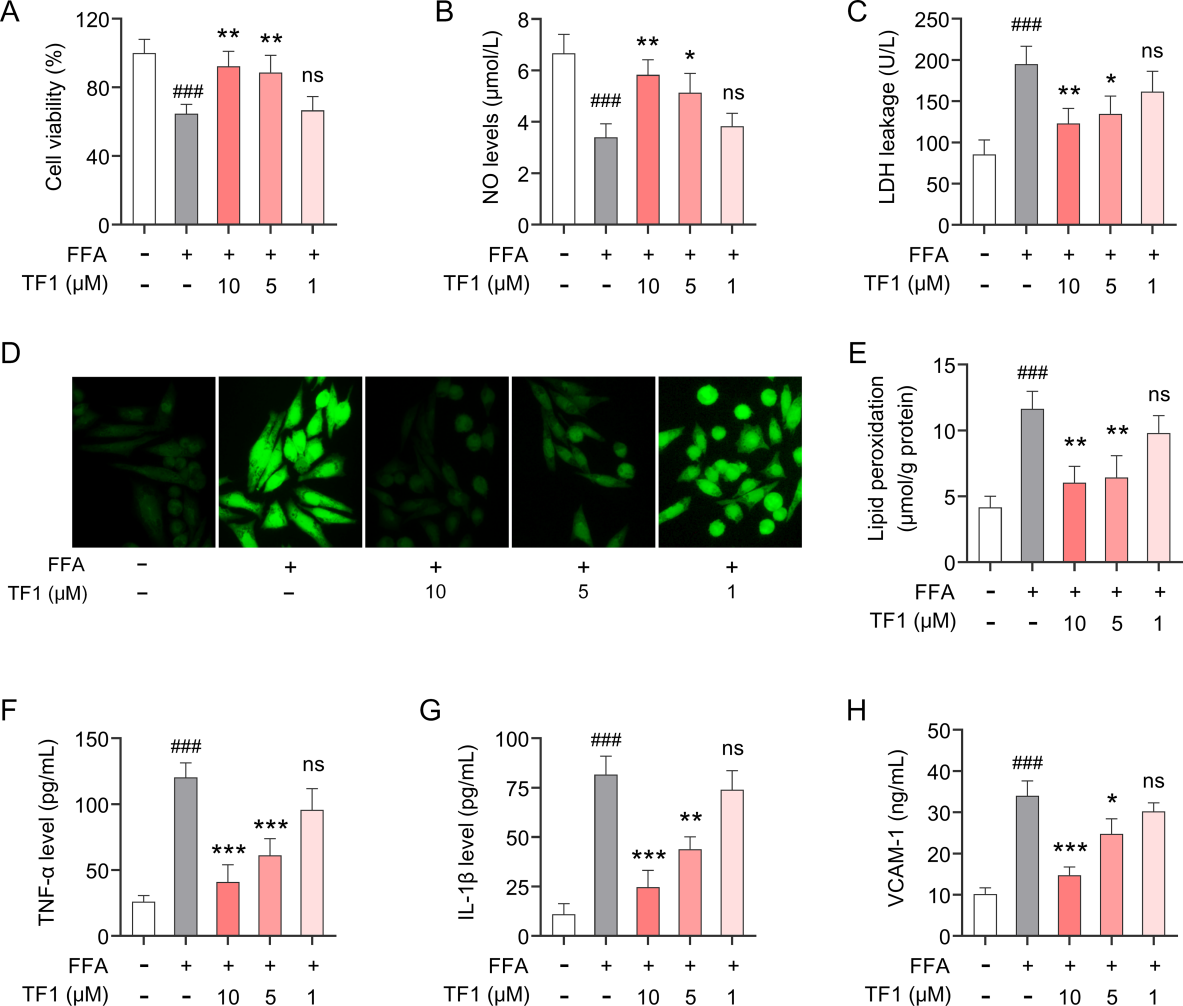
TF1 concentration-dependently protected EA.hy926 endothelial cells against FFA-induced injury, accompanied by reduced oxidative stress and inflammatory responses. **(A)** Cell viability of EA.hy926 cells following FFA exposure and TF1 pretreatment; **(B, C)** measurement of NO levels **(B)** and LDH leakage **(C)**; **(D)** fluorescent staining of intracellular ROS; **(E)** Intracellular lipid peroxidation; **(F–H)** the levels of TNF-α **(F)**, IL-1β **(G)** and VCAM-1 **(H)**. ^###^ p < 0.001 *vs.* Control group; ^*^p < 0.05, ^**^p < 0.01, ^***^p < 0.001, and ns (not significant) *vs.* FFA group. TF1, theaflavin; FFA, free fatty acid; NO, nitric oxide; LDH, lactate dehydrogenase.

Having identified TF1 as the lead candidate, we further characterized its concentration-response profile. TF1 exerted a clear concentration-response protective effect against FFA-induced cytotoxicity (**Figure 6A**). Treatment with 5 and 10 µM of TF1 significantly reversed the reduction in cell viability (p < 0.01), whereas the lowest concentration (1 µM) did not produce a statistically significant effect under the present conditions. This viability recovery was accompanied by normalized NO production (**Figure 6B**) and reduced LDH leakage (**Figure 6C**). At the same time, TF1 concentration-dependently reduced intracellular ROS generation (**Figure 6D**) and lipid peroxidation (**Figure 6E**). Furthermore, considering the crosstalk between oxidative stress and inflammation, we analyzed downstream inflammatory markers. TF1 treatment significantly abrogated the FFA-induced surge in pro-inflammatory cytokines (TNF-α, IL-1β) and the adhesion molecule VCAM-1 (**Figures 6F–H**). Together, these data showed that the protective effects of TF1 were accompanied by reductions in oxidative stress and inflammatory responses in a concentration-dependent manner.

### 3.7 Transcriptomics analysis revealed TF1 that the protective effects of TF1 was associated with regulation of the Nrf2-NF-κB axis

**Figure 7:**
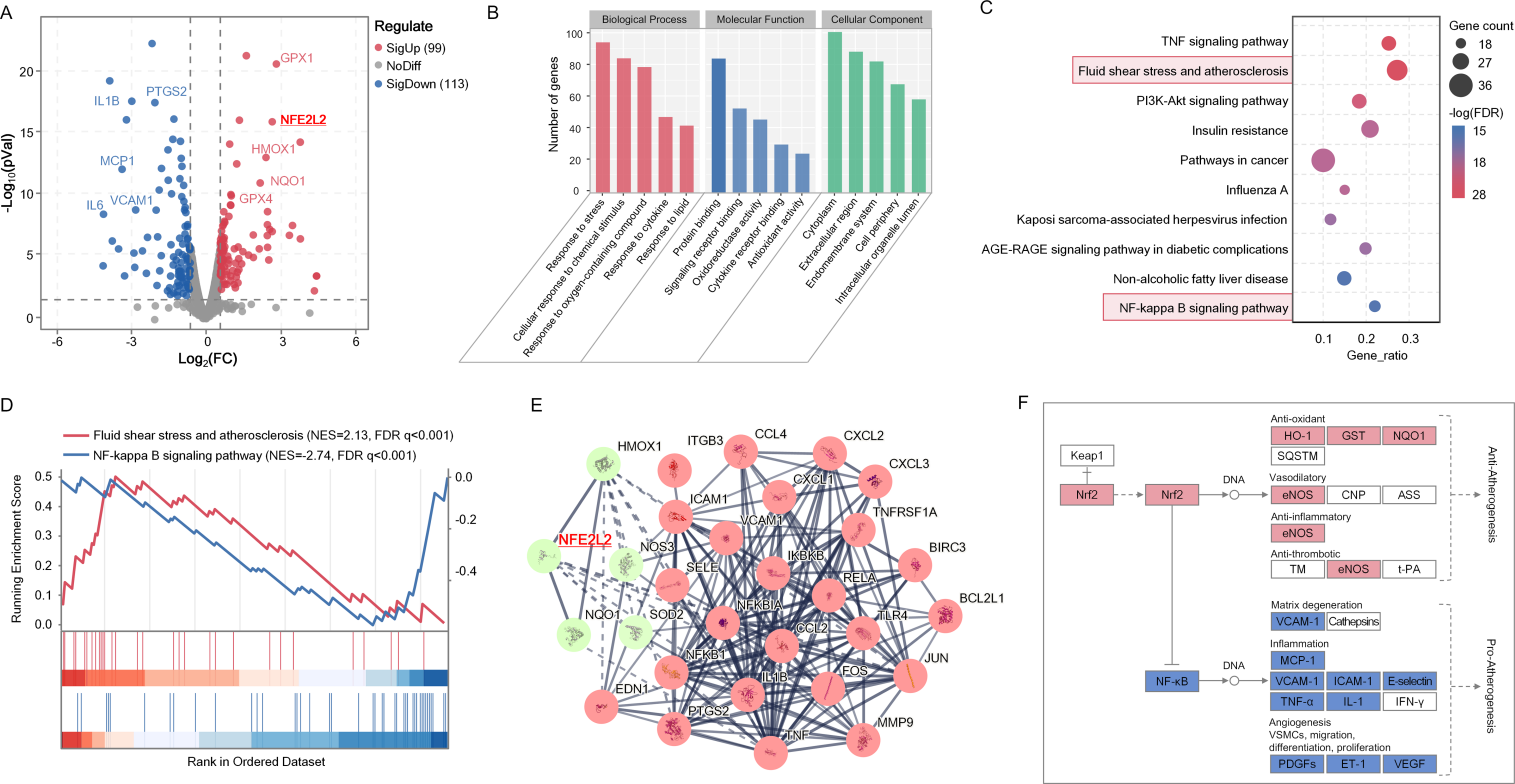
Transcriptomic analysis suggested that TF1 regulated Nrf2-NF-κB axis and alleviate FFA-induced injury in EA.hy926 cells. **(A)** Volcano plot illustrating DEGs induced by TF1; **(B)** GO annotation of DEGs across biological processes, molecular functions, and cellular components; **(C)** The top signaling pathways from KEGG enrichment analysis; **(D)** GSEA for fluid shear stress and atherosclerosis pathway and NF-κB signaling pathway; **(E)** PPI network of DEGs identifying NFE2L2 (Nrf2) as the central hub gene; **(F)** Integrated pathway map illustrating the regulation of Nrf2-NF-κB axis to cope with atherosclerosis. TF1, theaflavin; FFA, free fatty acid; DEGs, differentially expressed genes; GO, gene ontology; GSEA, gene set enrichment analysis; PPI, protein-protein interaction.

To systematically decode the molecular networks underlying the cytoprotective effects of TF1, we performed RNA sequencing on EA.hy926 cells. Differential expression analysis revealed that TF1 treatment induced a profound transcriptomic shift, with 99 genes significantly up-regulated and 113 genes down-regulated (**Figure 7A**). TF1 significantly upregulated key antioxidant regulators, including NFE2L2 (Nrf2) and its downstream effectors HMOX1 (HO-1), NQO1, and GPX1/4. Concomitantly, it potently suppressed key pro-inflammatory mediators such as IL1B, IL6, TNF, and VCAM-1. Furthermore, these DEGs were heavily enriched in clusters related to ‘Response to stress’ and ‘Oxidoreductase activity’ (**Figure 7B**). KEGG analysis underscored the involvement of these DEGs in pathways related to atherosclerosis and inflammation, such as fluid shear stress and atherosclerosis, and NF-kappa B signaling pathway (**Figure 7C**). GSEA results indicated that fluid shear stress and atherosclerosis pathway showed significant positive enrichment (normalized enrichment score (NES) = 2.13, FDR q < 0.001; **Figure 7D**). In contrast, NF-κB signaling pathway showed strong negative enrichment, indicating a broad suppression of this inflammatory hub. A protein-protein interaction (PPI) network identified NFE2L2 (Nrf2) as the central hub, connecting upregulated antioxidant genes and downregulated pro-inflammatory genes (**Figure 7E**). Based on refining the pathway map of fluid shear stress and atherosclerosis and NF-kappa B signaling pathway, a hybrid pathway was built to illustrate the potential involvement of the Nrf2-NF-κB axis in the protective effects of TF1 (**Figure 7F**). Overall, these transcriptomic data suggest that Nrf2 may represent an important regulatory node associated with the attenuation of oxidative-inflammatory injury by TF1 in FFA-induced EA.hy926 cells.

### 3.8 TF1 activated Nrf2-related antioxidant signaling and suppressed NF-κB-related inflammatory responses

**Figure 8:**
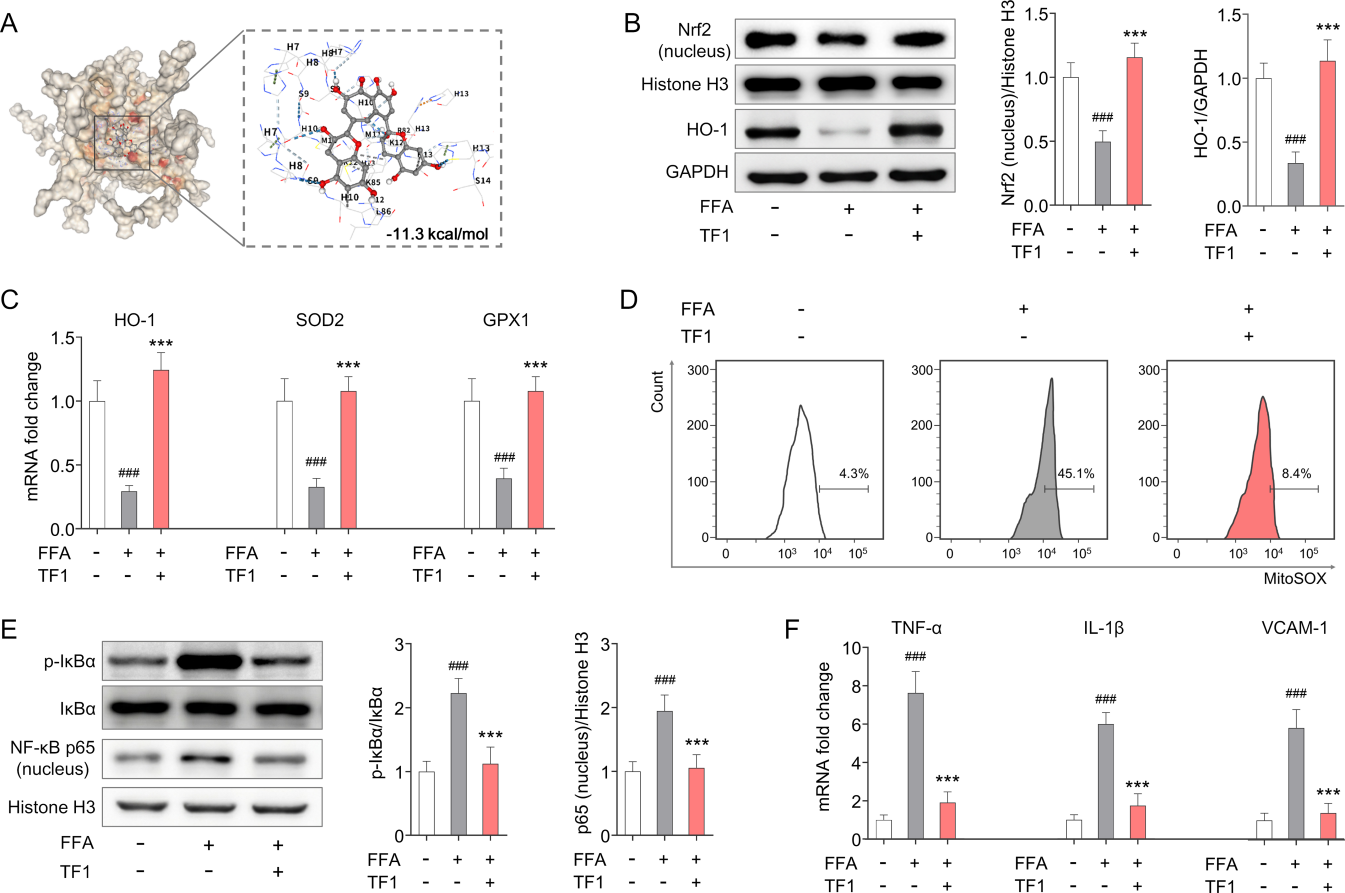
TF1 promoted Nrf2 nuclear translocation and was associated with activation of antioxidant defense and inhibition of NF-κB-driven inflammation in EA.hy926 cells. **(A)** molecular docking of TF1 with Keap1; **(B)** protein levels of nuclear Nrf2 and HO-1; **(C)** relative mRNA expression of antioxidant genes (HO-1, SOD2, GPX1); **(D)** flow cytometry analysis of mitochondrial ROS using MitoSOX red staining; **(E)** protein levels of p-IκBα and nuclear NF-κB p65 levels; **(F)** relative mRNA expression of pro-inflammatory genes (TNF-α, IL-1β, and VCAM-1). ^###^p < 0.001 *vs.* Control group; ^***^p < 0.001 *vs.* FFA group. TF1, theaflavin; FFA, free fatty acid; ROS, reactive oxygen species.

To experimentally validate the proposed mechanism, molecular docking was performed to investigate the potential interaction between TF1 and Keap1, the cytosolic repressor of Nrf2. The result revealed that TF1 showed multiple predicted interactions with Keap1 residues, with a binding energy of -11.3 kcal/mol (**Figure 8A**). suggesting the possibility that TF1 may interfere with the Keap1-Nrf2 interaction. Consistent with this result, Western blot analysis demonstrated that TF1 treatment significantly promoted the nuclear translocation of Nrf2 and increased the protein expression of its downstream effector, HO-1 (**Figure 8B**). At the transcriptional level, qPCR analysis confirmed that TF1 reversed the FFA-induced downregulation of anti-oxidative genes, including HO-1, SOD2, and GPX1 (**Figure 8C**). To assess the functional consequence of this antioxidant boost, we measured mitochondrial superoxide levels using MitoSOX staining. As shown in **Figure 8D**, FFA stimulation caused a massive accumulation of mitochondrial ROS, which was potently scavenged by TF. Given the well-recognized association between oxidative stress and NF-κB activation, we further examined the inflammatory signaling response. Western blotting showed that TF1 treatment effectively reduced IκBα phosphorylation and the nuclear accumulation of the NF-κB p65 subunit (**Figure 8E**). Consequently, the transcriptional expression of downstream pro-inflammatory mediators (TNF-α, IL-1β, and VCAM-1) was robustly suppressed by TF1 (**Figure 8F**). Together, these findings suggest that TF1 protected endothelial cells against lipotoxic injury in association with activation of Nrf2-related antioxidant defenses and inhibition of NF-κB-related inflammatory signaling.

### 3.9 TF1-mediated protection against FFA-induced endothelial injury depended on Nrf2

**Figure 9:**
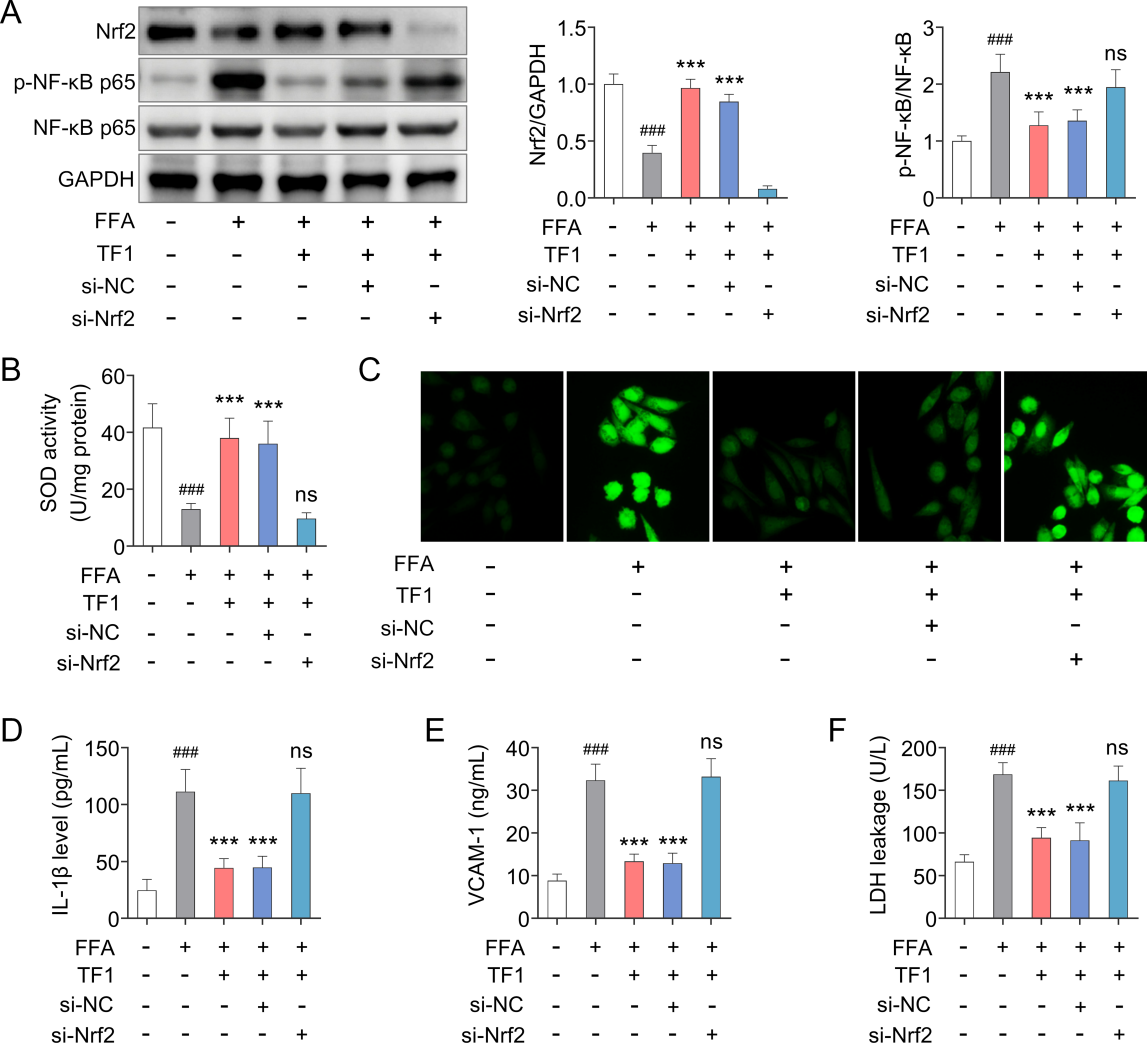
TF1-mediated protection against FFA-induced EA.hy926 cell injury depended on Nrf2. **(A)** Western blot analysis of Nrf2 protein levels to verify knockdown efficiency; **(B)** SOD activity; **(C)** intracellular ROS levels; **(D, E)** measurement of inflammatory markers IL-1β **(D)** and VCAM-1**(E)**; **(F)** LDH leakage assay to assess cell injury. ^###^p < 0.001 *vs.* Control group; ^***^p < 0.001, and ns (not significant) *vs.* FFA group. TF1, theaflavin; FFA, free fatty acid; SOD, superoxide dismutase; ROS, reactive oxygen species; LDH, lactate dehydrogenase.

To determine whether Nrf2 is essential for the protective effects of TF1, we performed knockdown experiments using Nrf2-specific siRNA (si-Nrf2). As shown in **Figure 9A**, si-Nrf2 transfection markedly reduced Nrf2 protein expression compared with the si-NC group, and TF1 failed to restore Nrf2 levels after silencing, confirming effective knockdown. Meanwhile, TF1 significantly inhibited FFA-induced NF-κB p65 phosphorylation, whereas this effect was markedly attenuated by Nrf2 silencing, as evidenced by the recovery of p-NF-κB p65 levels. These results indicated that Nrf2 was essential for the inhibitory effect of TF1 on NF-κB signaling. Furthermore, TF1 treatment effectively restored SOD activity and reduced ROS accumulation induced by FFA. However, these beneficial effects were blocked in Nrf2-knockdown cells (**Figures 9B, C**). Similarly, silencing Nrf2 eliminated the ability of TF1 to inhibit the secretion of inflammatory cytokines IL-1β (**Figure 9D**) and VCAM-1 (**Figure 9E**). Consequently, LDH leakage in the TF1 + si-Nrf2 group was not significantly different from that in the FFA group, suggesting that the protective effect of TF1 on endothelial injury was largely abolished after Nrf2 silencing (**Figure 9F**). Collectively, these results demonstrated that Nrf2 was required for TF1 to exert its therapeutic effects.

## 4 Discussion

Elevated circulating FFAs constitute the fundamental pathogenic soil for the development of cardiovascular and metabolic diseases. In vascular endothelial cells, NO is a central homeostatic mediator that preserves endothelial function by promoting vasodilation and suppressing platelet activation, leukocyte adhesion, and vascular inflammation. Therefore, reduced NO bioavailability is widely recognized as a hallmark of endothelial dysfunction. In the vascular endothelium, FFA-induced oxidative stress disrupts NO bioavailability, primarily through excessive ROS generation, which can directly quench NO and impair endothelial redox homeostasis, thereby contributing to endothelial activation and inflammatory responses that may favor the development of atherosclerosis (11). In the liver, lipid overload drives the transition from simple steatosis to non-alcoholic steatohepatitis (NASH) through lipotoxic hepatocellular injury (3). Therefore, targeting the dysregulated oxidative-inflammatory axis has emerged as a promising therapeutic strategy for alleviating FFA−induced lipotoxic injury. Black tea consumption has long been associated with metabolic resilience, and its beneficial effects have been primarily attributed to the abundant TFs (21, 22). However, the precise contribution of individual TFs has remained obscured. Although current studies on black tea or TFs have often focused on their capacity to reduce lipid accumulation (23, 24), our results showed that TFs did not significantly alter intracellular lipid droplet accumulation or FFA levels in endothelial and hepatic cells under the present experimental conditions. Importantly, lipid droplets are dynamic, tightly regulated organelles rather than inert lipid stores. Because their formation and turnover are governed by multiple pathways, overall lipid measurements may not fully reflect changes in lipid droplet biology. Thus, the absence of major changes in intracellular lipid accumulation does not exclude a potential effect of TFs on lipid droplet dynamics. Our data instead suggested that, under the conditions tested, TFs primarily alleviated lipotoxicity through modulation of the FFA-ROS-inflammation axis, without markedly reducing overall lipid accumulation. Notably, despite the distinct functional characteristics of endothelial and hepatic cells, the two models showed a broadly similar response pattern, in which TFs exerted limited effects on overall lipid accumulation but consistently attenuated oxidative stress- and inflammation-associated damage.

By performing systematic comparisons among the four primary subtypes (TF1, TF2A, TF2B, and TFDG), our study suggests a bioactivity hierarchy under the present experimental conditions. Our side-by-side comparison revealed that TF1 exhibited the strongest bioactivity in cellular models of lipotoxicity, outperforming the mono-galloylated (TF2A/B) and di-galloylated (TFDG) forms. This finding highlights a notable divergence between chemical radical-scavenging capacity (often measured in cell-free assays) and intracellular biological efficacy, suggesting that the antioxidant capacity of TFs does not necessarily correlate positively with the number of galloyl moieties. Antioxidant assays in both cell-free systems and H_2_O_2_-stimulated cells indicated that TFs, particularly TF1, exerted antioxidant effects not only through direct radical scavenging but also, more importantly, via activation of the endogenous cellular antioxidant system. The H_2_O_2_ model was included as a complementary oxidative stress model to assess the antioxidant effects of TFs under a simplified ROS-driven condition. In this model, H_2_O_2_ increased ROS accumulation and reduced SOD and GPx activities, accompanied by decreased SOD2 and GPX1 expression, indicating impairment of endogenous antioxidant defense. TF1 most effectively reversed these changes, supporting its superior ability to reinforce cellular antioxidant capacity. The direct scavenging capacity of TFs is correlated with their hydroxyl-rich frameworks that facilitate hydrogen atom transfer to neutralize free radicals, a process governed by hydroxyl bond dissociation energy (BDE) (25, 26). The superior radical-scavenging activity of TF1 and TFDG may be attributed to the relatively low BDE of their hydroxyl groups. Notably, TF1 exhibited greater intracellular antioxidant efficacy than TFDG. This difference may be related to its more favorable steric configuration, which could facilitates interaction with key antioxidant regulatory proteins, compared with the bulkier and more sterically hindered TFs (TF2A/B and TFDG) (27). Together, these findings support the interpretation that FFA impairs endogenous antioxidant defense and that TF1 counteracts this effect.

**Figure 10:**
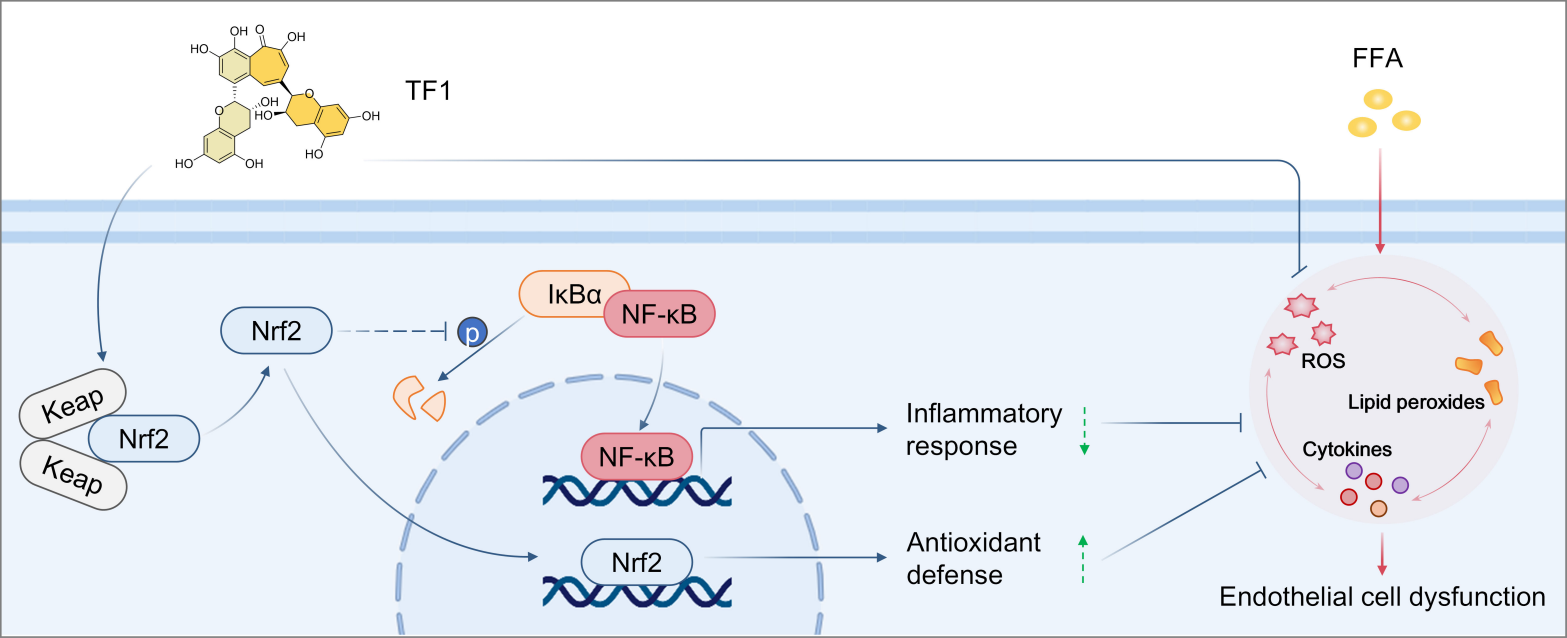
Schematic diagram of mechanism underlying the protective effects of TF1 against FFA-induced endothelial dysfunction. TF1, theaflavin; FFA, free fatty acid; ROS, reactive oxygen species.

Mechanistically, our data suggest that the Nrf2-NF-κB axis is an important pathway associated with the protective effects of TF1 under lipotoxic conditions. The Nrf2 signaling pathway serves as a critical defensive mechanism against oxidative injury. Upon activation, Nrf2 translocates to the nucleus and stimulates the transcription of downstream antioxidant enzymes, thereby defending against oxidative stress from multiple sources (28–31). The crosstalk between oxidative stress and inflammation is well-documented, where ROS acts as a secondary messenger to activate the NF-κB pathway and cytokine secretion, such as in both atherosclerosis and MASH (32–35). While Zeng et al. previously reported that theaflavins could activate Nrf2 to alleviate oxidative injury (18), our study extends this knowledge by identifying TF1 as the most active monomer in our models and suggesting its role in attenuating the oxidative stress-inflammation loop.TF1 restored the redox balance via Nrf2-mediated upregulation of phase II enzymes (HO-1, SOD2), accompanied by reduction of ROS accumulation and suppression of NF-κB-related inflammatory signaling, thereby uncoupling the pathogenic feedback loop between oxidative stress and inflammatory response. Notably, restoration of redox homeostasis in endothelial cells was accompanied by recovery of NO production, indicating that TF1 improved endothelial functional status. Nrf2 silencing markedly weakened the protective effects of TF1, confirming that Nrf2 is required for the protective effects of TF1. This hierarchical regulation explains how TF1 can suppress oxidative stress and synchronously inhibit the expression of pro-inflammatory cytokines (TNF-α, IL-1β) and the adhesion molecule (VCAM-1) without major changes in the measured lipid accumulation parameters, effectively arresting the transition from metabolic stress to endothelial dysfunction (**Figure 10**). Since LDH leakage is an early pathological event in atherosclerosis (36), and the induction of adhesion molecules is a rate-limiting step in atherosclerotic plaque formation (37), the ability of TF1 to fortify the endothelial barrier against oxidative erosion suggests a potential to arrest the disease at its initiation phase.

Nevertheless, this study has several limitations. First, the conclusions are derived mainly from *in vitro* endothelial and hepatic cell models; therefore, *in vivo* validation is required to determine whether the protective effects and bioactivity hierarchy of individual TFs can be reproduced in more complex metabolic settings. Second, although we assessed representative indicators related to cell injury, oxidative stress, and inflammatory responses, the current panel of endpoints remains relatively limited. Third, although 10 μM TF1 showed clear protective effects *in vitro*, this concentration may exceed typical circulating levels achieved through normal dietary intake because of the limited contents and bioavailability of tea polyphenols. Thus, it should be regarded primarily as a mechanistically relevant *in vitro* dose rather than a direct reflection of systemic exposure *in vivo*. Future studies incorporating animal models, together with broader mechanistic and functional indicators, will be important to further substantiate the biological relevance and translational potential of TF1.

## 5 Conclusion

This study demonstrated that TFs effectively attenuated oxidative stress and inflammatory responses, thereby interrupting the self−perpetuating oxidative-inflammatory cascade that underlies FFA-induced endothelial dysfunction and hepatic injury in metabolic and vascular disorders. Among the tested TF subtypes, TF1 consistently exhibited the most pronounced cytoprotective efficacy across both cell types, suggesting its cross−tissue protection at key metabolic interfaces. Mechanistically, TF1 exerted the protective actions predominantly through coordinated activation of the Nrf2−dependent antioxidant defense system and suppression of NF−κB-mediated pro−inflammatory signaling. This dual modulation restored redox homeostasis and limited inflammatory amplification under lipotoxic conditions, positioning the Nrf2-NF−κB axis as a central molecular pathway associated with TF1 action. These results advance mechanistic understanding of theaflavin subtype–specific bioactivities and underscore their potential as safe, dietary−derived modulators for preventing or alleviating FFA−driven vascular and hepatic complications.

## Data Availability

The original contributions presented in the study are included in the article/**Supplementary Material**. Further inquiries can be directed to the corresponding author.
