# Supplementary material for: Theaflavins counteract free fatty acid-driven oxidative-inflammatory injury in endothelial cells through Nrf2-NF-κB axis
Source: Front Immunol. 2026 Jun 9;17:1807168. doi: 10.3389/fimmu.2026.1807168 (PMC13286788; doi:10.3389/fimmu.2026.1807168)
Supplement: Supplementary file 1 [file Table1.docx]

Supplementary Material

**Supplementary Table S1**. Primer sequences used in Real-time PCR experiment

| **Primer** | **Forward** | **Reverse** |
| --- | --- | --- |
| HO-1 | 5′-CCAGGCAGAGAATGCTGAGTTC-3′ | 5′-AAGACTGGGCTCTCCTTGTTGC-3′ |
| SOD2 | 5′-GCCTGCACTGAAGTTCAATG-3′ | 5′-ATCTGTAAGCGACCTTGCTC-3′ |
| GPX1 | 5′‑CAGTGGGCATCAGGAAGATC‑3′ | 5′‑TCTCCTGATGTCCCAGGAAA‑3′ |
| TNF-α | 5′-TGAAAGCATGATCCGGGACG-3′ | 5′-TGAGGTACAGGCCCTCTGAT-3′ |
| IL-1β | 5′-ATGATGGCTTATTACAGTGGCAA-3′ | 5′-GTCGGAGATTCGTAGCTGGA-3′ |
| VCAM-1 | 5′‑CCTGAAGATGGATGCAGTGA‑3′ | 5′‑GGTCTCCTCAGCTTCAGTCA‑3′ |
| GAPDH | 5′-GAAGGTGAAGGTCGGAGTCAA-3′ | 5′-CTTCCCGTTCTCAGCCATGTA-3′ |

**Supplementary Table S2**. Sequences for siRNA transfection

| **Gene** | **Sense** | **Antisense** |
| --- | --- | --- |
| si-Nrf2 | 5′-GCCTTTTTCGCTCAGTTACAA-3′ | 5′-TTGTAACTGAGCGAAAAAGGC-3′ |
| si-NC | 5′-AATGTGCCGGTGGCAGGCTTA-3′ | 5′-TAAGCCTGCCACCGGCACATT-3′ |
